# Sustainable Approaches Using Green Technologies for Apple By-Product Valorisation as A New Perspective into the History of the Apple

**DOI:** 10.3390/molecules27206937

**Published:** 2022-10-16

**Authors:** Rocío De la Peña-Armada, Inmaculada Mateos-Aparicio

**Affiliations:** Departamento de Nutrición y Ciencia de los Alimentos Facultad de Farmacia, Universidad Complutense de Madrid, Plaza Ramón y Cajal s/n, 28040 Madrid, Spain

**Keywords:** apple, apple by-product, valorisation, green technologies, bioactive compounds

## Abstract

The apple has been recognised as the most culturally important fruit crop in temperate land areas. Centuries of human exploitation and development led to the production of thousands of apple cultivars. Nowadays, the apple represents the third most widely cultivated fruit in the world. About 30% of the total production of apples is processed, being juice and cider the main resulting products. Regarding this procedure, a large quantity of apple by-product is generated, which tends to be undervalued, and commonly remains underutilised, landfilled, or incinerated. However, apple by-product is a proven source of bioactive compounds, namely dietary fibre, fatty acids, triterpenes, or polyphenols. Therefore, the application of green technologies should be considered in order to improve the functionality of apple by-product while promoting its use as the raw material of a novel product line. The present work provides a holistic view of the apple’s historical evolution, characterises apple by-product, and reviews the application of green technologies for improving its functionality. These sustainable procedures can enable the transformation of this perishable material into a novel ingredient opening up new prospects for the apple’s potential use and consumption.

## 1. Introduction

**Figure 1:**
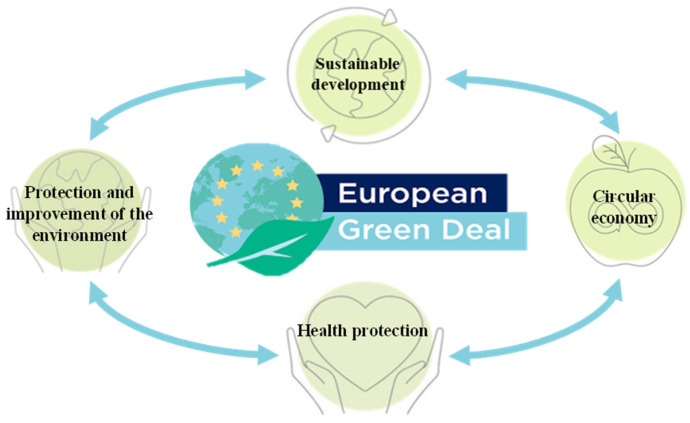
Graphical representation of certain fundamental issues for the European Green Deal.

In September 2015, the General Assembly of the United Nations established the 17 Sustainable Development Goals (SDGs) for the 2030 Agenda for Sustainable Development. The defined goals are intended to generate a worldwide impact on the ecological, social, and economic spheres. Among the stated objectives, Goal 12: Responsible Consumption and Production, is focused on ensuring sustainable consumption and production patterns [1,2]. Along the same line, the European Commission developed Europe’s new agenda for sustainable growth, known as the European Green Deal. In fact, it is a contemporary strategy which aims to transform the European Union into a fair and thriving society, where economic progress is dissociated from resource usage. Under the EU Green Deal, the Circular Economy Action Plan aims to promote the sustainable use of European resources along the entire life cycle of products, and therefore, protect natural ecosystems and improve human health (Figure 1) [3].

It is estimated that one-third of global food production for human consumption is lost or wasted. These data correspond to approximately 1.3 billion tonnes of food, which are currently discarded, from primary production up to consumption [4]. *Food loss* has been defined by the Food and Agriculture Organization of the United Nations (2019) as “the decrease in quantity or quality of food along the food supply chain, from harvest/slaughter/catch up to, but not including the retail level”. On the other hand, *food waste* occurs at the retail and consumption levels [5]. Overall, developing countries account for the greatest loss during production and postharvest (75% of food losses). Besides, for industrialised countries, waste mainly occurs in the consumption stage (more than 40% of food waste) [6,7]. Particularly, European countries generated 96 million tonnes of vegetables and fruits in 2017, and around 29 million tonnes were thrown away as waste [4]. The main losses of fruits and vegetables are due to agricultural production, mostly related to post-harvest grading, caused by settled quality standards [7].

Food loss and waste have a significant impact on food quality, security, and safety, as well as on natural resources and environmental protection [8]. Traditionally, agro-industrial leftovers were disposed of in landfills or incinerated with the subsequent pollution and contamination effect [9]. Therefore, co-product and by-product management has gained increasing attention in the food science and food industry fields. In fact, there is a growing interest, reflected in the scientific literature, in the application of treatment methods for food by-product valorisation [8]. In addition, the recent trend of by-product transformation into added-value products includes the application of eco-efficiency, and cleaner and greener processes [10]. The ultimate goal is to reuse and recycle these by-products in order to return them to the production chain [8]. Thus, circularity is promoted, enabling the closing of the loop and decreasing the amount of discarded materials disposed of in the environment [11].

Food by-products are considered non-edible portions of agri-food products. In addition, they can contain a significant amount of bioactive compounds with high value and a wide range of applications such as food additives or nutraceuticals [12]. According to Ziun and Ramin [10], over the last years, the most commonly studied raw materials for the recovery of natural products through green and sustainable methods were fruits, grains, and other abundant materials [10]. In this context, it seems remarkable to investigate one the most globally consumed fruits, which is the apple. 

The apple represents the fourth most important fruit eaten around the world and comprises a harvested area of 4.7 million hectares worldwide [13,14]. Thousands of apple cultivars are grown to produce an ample variety of apples for the fresh market, and also a broad range of other food products [15]. A significant amount of apples is discarded along the food chain supply, including harvesting and processing stages. However, apple by-product may be utilised for both, recovering their valuable compounds and developing new products with added value [16]. This major step forward opens a new dimension into the life cycle of the apple, promoting the development of innovative products while improving the quality of the environment. 

To the best of our knowledge, there are no approaches in the literature providing a holistic view of the apple’s history that deepen into the present challenges of addressing apple waste. Therefore, the aim of the present work is to understand the historical evolution of the apple, characterise apple by-product, and review current strategies for apple by-product valorisation. Thus, this emblematic fruit, as it has been since ancient times, will be consumed fresh or processed, but the resulting apple by-product could achieve its full potential as a novel food ingredient. The fundamental idea is to seize every opportunity to valorise this source of bioactive compounds. 

## 2. History of the Apple

**Figure 2:**
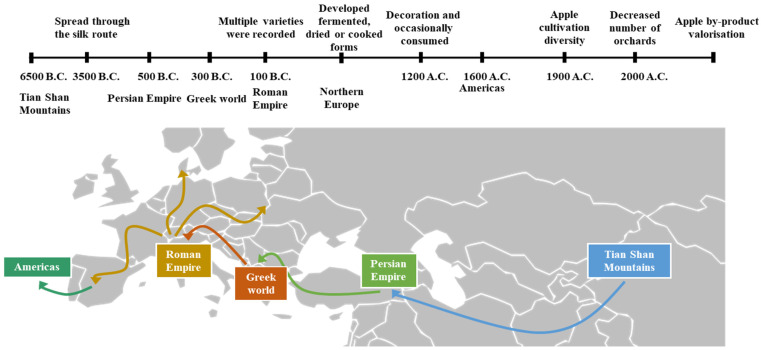
Evolutionary history of the cultivated apple.

The apple tree (*Malus domestica* Bork) has been a symbolic element throughout human history and traditionally occupies a central position in culture, art, and folklore. *Malus domestica* belongs to the Rosaceae family. The taxonomy of the *Malus* genus consists of about 30 species and numerous subspecies. This cultigen was domesticated from *M. sieversii*, described as the primary progenitor, in the Tian Shan Mountains (6500 B.C.), and diffused through the Silk Road (Figure 2) from Central Asia to West Europe [17,18]. By 500 B.C., the apple was probably dispersed through the Persian Empire, and around 300 B.C., when Alexander the Great conquered the Persians, apple cultivation geographically expanded through the Greek world. Subsequently, the Roman Empire promoted the cultivation of the domesticated apple, probably hybridised with the native crab apple (*M. sylvestris*), through west and north Europe. Furthermore, cider became popular in the Greek and Roman worlds. However, this beverage was not comparable to wine. Nevertheless, cider became associated with areas where grapes were not successful, corresponding to northern regions of Europe. The rise of Christianity in the following centuries favoured the maintenance of apples throughout Europe. From the 13th century on, apples were widely planted throughout Europe in the gardens of royalty. Furthermore, apples were consumed, occasionally raw but also cooked, blended with honey, sugar or spices, or as fermented juice. During the 16th and 17th centuries, the European colonists brought the apple to the Americas. In the late 19th and early 20th century, apple cultivation diversity in Europe reached its apogee with thousands of small orchards and hundreds of local cultivars. In the 20th century, the rise of imported fruit from different continents forced orchards to increase in size and decrease in number. Therefore, by the 21st century, apple cultivation diversity has been greatly reduced [15,18,19].

Centuries of human exploitation and selection led to the production of numerous apple cultivars making this cultivation the major fruit crop in temperate zones [20,21]. Thus, the apple is currently the third most produced fruit in the world (87.2 million tonnes/year), after the banana (116.8 million tonnes/year), and watermelon (100.4 million tonnes/year) fruits [14]. It is estimated that 40 apple varieties account for the greatest proportion of commercial production, and particularly “Delicious”, “Jonagold”, and “Gala” have a major impact on the market. Besides, differences from country to country regarding the relative importance of apple cultivars are evident, namely, southern Europe produces much “Golden Delicious”, whereas in Northern Europe the “Elstar” and “Jonagold” varieties are the most popular. Australia and New Zealand constitute the major exporters of “Gala”, “Granny Smith”, and “Braeburn” apples. For the Republic of China, the leading cultivar is “Fuji”, whereas in many regions of North America, “McIntosh” and “Delicious” are predominant [18,22]. Fresh apple consumption has been and still is the primary market for most apples produced around the world. Domestic fresh use accounts for approximately 60–75% [18]. On the other hand, 20–40% of total world production is processed and converted into numerous products, including juices, cider, jams, or vinegar. Apple juice and cider are the most remarkable products, with apple juice being 65% of the total amount of processed apples [23,24,25,26,27,28]. At the same time, cider is gaining a lot of popularity among actual society, and the total revenue is expected to grow at a 6.5% compound annual growth rate from 2021 to 2027 [29]. Hence, along the industrial food chain, from primary production to processing, there is 25% of the apple mass, referred as apple by-product, which is discarded as waste [30].

Although apple by-product is considered a source of bioactive compounds, presently, it remains underutilised after apple processing by the food industry, and it is usually intended to landfill as fertiliser or as animal feed [31]. However, the utilisation of apple by-product has been investigated in the last decade, and significant beneficial usages have been determined [32]. Among them, the development of bioactive ingredients or novel food additives from apple waste is of great interest [33,34]. 

## 3. Apple By-Product

**Figure 3:**
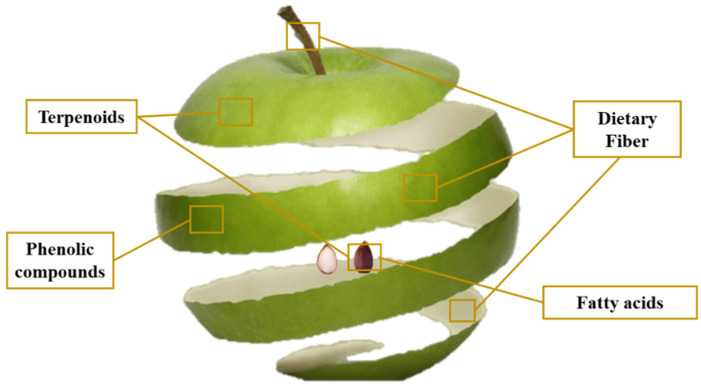
Apple by-product as a source of terpenoids, phenolic compounds, fatty acids, and dietary fibre.

Apple by-product could be defined as the remaining solid of apple processing in order to obtain juices or cider, among other products. It is mainly constituted of apple peel, seeds, stems, and pulp. Regarding the proximate composition of apple by-product (Table 1), the main element, overall, is dietary fibre, followed by non-fibrous carbohydrates, lipids, proteins, and ashes. Apple by-product has been recognised as a health-promoting ingredient by numerous authors (Table 2) and due to its composition, it has been determined as a potential source of bioactive compounds, such as dietary fibre, and also phenolic compounds, triterpenoids, or fatty acids (Figure 3) [24,30,35]. Polyphenolic antioxidants in apples are predominantly located in the skin, thus, most polyphenols persist in the apple pomace [30]. Phenolic compounds found in apple by-product include flavanols as the major class of apple polyphenols, followed by hydroxycinnamic acid derivatives, flavonols, di-hydrochalcones, and anthocyanins [24]. They have exhibited a high scavenging activity, indicating apple by-product as a potential source of dietary antioxidants [36]. Besides, triterpenic acids are abundant in many plants, including apples [37]. Particularly, apple terpenoids might be found either in the peel epidermal cells or epicuticular wax, which are often discarded as food processing industry waste [38,39]. Triterpenic acids have demonstrated various pharmacological properties and health effects, such as antioxidant, anti-inflammatory, or antimicrobial effects, increasing the attraction to producing healthcare products with multifunctional values [37,40]. Regarding fatty acids, recent studies have verified the presence of unsaturated fatty acids in the apple seed and apple peel. Particularly, oleic, linoleic, and linolenic acids have been described as the main components of this lipid fraction [35,41]. The contribution of the fatty acids to the potential antioxidant, antimicrobial, and antiproliferative capacities of apple seed oil has been proved [42,43,44].

Dietary fibre represents the highest contribution in the proximate composition of apple by-product (55.48 ± 0.7 g/100 g dry matter). Insoluble dietary fibre (IDF) accounts for the greatest proportion (43.58 ± 0.6 g/100 g dry matter) whereas soluble dietary fibre (SDF), represents a lower percentage (11.06 ± 0.1 g/100 g dry matter) [45]. Besides, apple by-product’ dietary fibre is mainly constituted of non-starch polysaccharides, including a high range of molecular sizes, structures, and monomeric composition. De la Peña-Armada and collaborators [60] determined the configuration of the total dietary fibre indicating uronic acids and glucose as principal components. In addition, arabinose, xylose, and galactose were also remarkable, suggesting the presence of pectin substances made of arabinans, arabinoxylans, homogalacturonans, arabinogalactans, or galactans as side chains. 

Pectins are present in the cell walls and intracellular tissues of fruit and vegetables [61]. They are mainly composed of numerous chains of galacturonic acid and are characterised by being resistant to human digestive enzymes. Pectins can be fermented along the intestine favouring the growth of *Lactobacillus or Bifidobacteria*, therefore promoting the release of acetate and propionate, and enhancing gut health [62,63]. In fact, apple by-product potential as a prebiotic ingredient has been demonstrated and mainly attributed to its pectin content [33,34,45,62]. 

Gibson and collaborators [64] proposed the following definition of a prebiotic: “*A substrate that is selectively utilised by host microorganisms conferring a health benefit.*” Certain fermentable carbohydrates have been described to promote a prebiotic effect. However, the most widely documented dietary prebiotics are the non-digestible oligosaccharides fructans and galactans [65]. Prebiotics have the potential to improve digestive function, modulate the immune system, improve mineral absorption, or help glucose metabolism. Furthermore, prebiotic compounds can promote metabolic health, including preventing insulin resistance and promoting healthy blood lipid levels [66,67]. The main derived products of bacterial prebiotic metabolism are the short-chain fatty acids, namely, acetate, butyrate, and propionate. These compounds are known to interact with the host systems, leading to health benefits in the gut microbial community and consequently elsewhere in the body. Thus, short-chain fatty acids have been suggested as an interesting strategy for gastrointestinal dysfunction, obesity, and type 2 diabetes mellitus prevention [68]. Besides, the feasibility of recovering prebiotic substrates from novel sources, such as food by-product, has been recently proposed for addressing the economic and environmental current needs [69].

Therefore, efforts of the juice and cider industry should be focused on the valorisation of the apple by-product for the development of functional foods, dietary supplements, and/or food additives [34]. 

## 4. Valorisation Procedures

**Figure 4:**
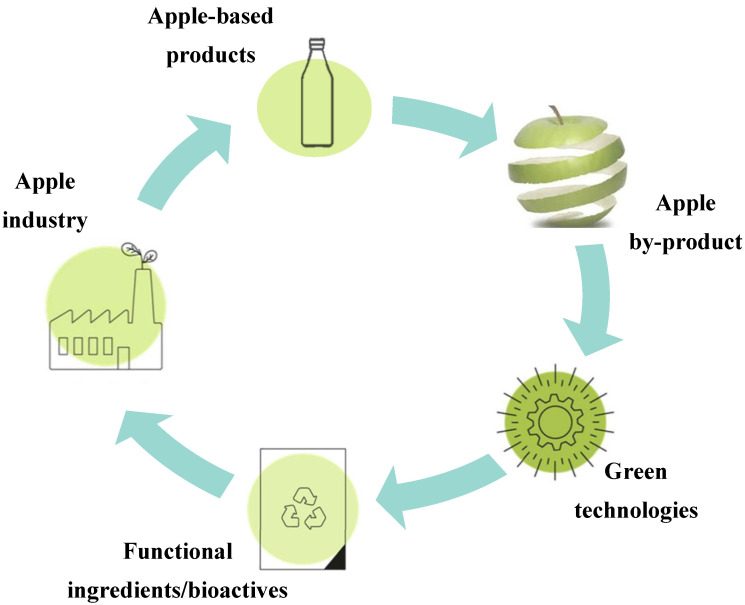
Apple industry based on green and sustainable technologies in the scope of circular economy.

Sustainable chemistry has suggested that food by-products are rich sources of bioactive compounds [70]. The convenience of improving this raw material’s functionality, namely, increasing the accessibility to the health-promoting components, suggests the possibility to design different transformation strategies. The enhancement of the nutritional value of by-products is of great relevance due to its human health, economic, and environmental benefits [16]. In this regard, green processes are based on methodologies with low energy consumption, use Generally Recognised as Safe (GRAS) solvents, and ensure a high-quality and safe product [71]. The main objective of those procedures consists of developing new natural resource products with added value while decreasing waste generation. Emerging technologies, such as ultrasound, enzyme digestion, or pulsed electric field, have been developed over the last fifty years. However, these innovative alternatives have been greatly implemented in the food, pharmaceutical, and cosmetic sectors [71,72]. Indeed, numerous studies are focused on the application of microwave, ultrasound, enzymes, high hydrostatic pressure, and supercritical fluid extraction, among others, for food by-product valorisation (Figure 4) [72].

Microwave extraction has developed an important role in food science and technology over the last two decades. Microwaves generate heat after their contact with cellular polar compounds. The heat disrupts the cell walls and therefore enhances the availability of certain components [73]. Microwave-assisted extractions are generally performed in polar media such as water, acetone, alcohol, or mixtures. The reaction typically requires between seconds and a few minutes [74]. Furthermore, microwaves have been studied in combination with different processing technologies. In fact, the microwave-assisted ultrasound extraction technique has been described as a novel method for fast and efficient extraction. It has great potential for commercial applications due to the fast sample preparation and the appropriate balance between costs and effectiveness [75]. On the other hand, ultrasound is based on sound waves, i.e., mechanical vibrations, which, when applied to plant-based matrices, can collapse cavitation bubbles close to cell walls, producing a cell disruption. Thus, this methodology enhances the extraction processes [73]. The spectrum of ultrasonic wave frequency varies in the range from 20 kHz, considered the audible range, up to 10 MHz [76].

Enzyme-assisted extraction methods are becoming increasingly popular [77]. Enzymes, which can be found in nature, have been used for thousands of years to produce different food products. However, the latest development within biotechnology enables the creation of tailor-made enzymes, which display new activities, and can be adapted to diverse process conditions [78]. This enzymatic non-conventional methodology provides the opportunity of processing foods in a short time and at a low temperature, achieving high extraction yield in the industry [79]. The substrate specificity favours the extraction of a great number of bioactive compounds from the food matrix [77]. Therefore, according to the desired results after the application of this methodology, the enzyme utilised for the procedure will vary. For instance, if the main goal is cell wall disruption, degradation is generally carried out by complex cellulolytic enzyme preparations, which contain a mixture of several cellulase kinds [80]. Thus, inaccessible bound forms, contained within cellular walls, can be released and their availability increased [77]. Enzyme-assisted extraction is usually considered within the so-called bioprocessing. Bioprocessing includes numerous methodologies in which microbial fermentation is notable. Fermentation requires the contribution of living microorganisms for the generation of new products. In addition, improves organoleptic properties, increases the product shelf-life and modifies the nutritional profile of food. The fermentation valorisation process has been demonstrated to be a feasible method to improve bioactive component content in food by-products [81,82,83,84,85].

High hydrostatic pressure (HHP) treatment is a cold pasteurisation technique in which products are subjected to a high level of isostatic pressure transmitted by water. The application of this technology destroys pathogens, extends product shelf-life and only requires water and electricity. Furthermore, numerous studies have concluded that HHP is not only a conservation technology but also enables the extraction of bioactive compounds [74,86]. HHP may increase the extractability of phenols, carotenoids, and anthocyanins, and also the bioavailability of minerals, antioxidants, and starch [74]. Besides, HHP could be relevant for food by-product valorisation, since these derived products have been over-processed, and further treatments, especially thermal procedures, could cause an excessive loss of their bioactive compounds’ functionality [87]. Additionally, HHP has been combined with different methodologies, including the incorporation of food-grade enzymes into the process for a potential application in the enhancement of the health benefits of food products [86,88]. Particularly, HHP assisted by certain enzymes has resulted in an increase in the enzymatic activity that may be due to a change in the active site or in substrate specificity [89].

Another alternative green process for food by-product valorisation is supercritical fluid extraction (SFE). The supercritical technique has been used since 1882 and its main application fields are food and flavour, pharmaceuticals, and various biochemical sectors [90]. Supercritical carbon dioxide enables a clean extraction to separate different components from food matrices, producing a pure and safe product [91,92]. Particularly, CO_2_ is a non-polar solvent which facilitates lipophilic compound obtention [42,93]. In addition, this procedure has other major advantages, such as the low critical temperature required for it to be conducted, or the absence of light and air during the process, which reduces the risk of recovered compound degradation [94]. 

Emerging technologies present various applications forms, including small-scale and large-scale, entailing industrial uses, such as food or nutraceutical processing [77]. Hence, in order to reintroduce the apple by-product back into the food chain, these technologies represent an interesting option for the present and future time.

## 5. Innovation for Apple By-Product Valorisation

It is known that apple by-product, despite its bioactive compounds, is used for animal feed or to extract pectins, although generating another waste in the latter. Indeed, it is estimated that only a fifth of the produced apple pomace is used for animal or human feed [95]. The high perishability of apple by-product is likely to be a major constraint for the effective utilisation of this derived product. Hence, it commonly requires a high cost- and energy- drying step for preservation, which also may modify sensorial characteristics and nutritional value of the fresh by-product, causing the loss of interesting components such as phenolic compounds [23,33,96].

Certain uses of dried apple by-product have been described in the literature, for instance, apple by-product added in stirred-type yoghurts and yoghurt drinks as a natural stabiliser as well as a dietary fibre source. Furthermore, apple pomace used to formulate biscuits reduced the resulting product’s glycaemic index [97,98]. In addition, this by-product has been incorporated into meat products such as chicken sausages achieving a dietary fibre-enriched and organoleptically acceptable product [99]. On the other hand, the applications of chemical, physical, or enzymatic procedures have demonstrated to be an appealing option to improve the apple by-product functional features, [22,40].

The application of green technologies, considered environmentally friendly, has proved to be a suitable alternative for food-derived product valorisation. These methodologies may increase the bioaccessibility of the diverse health-promoting compounds, including dietary fibre, fatty acids, and antioxidants [22,23]. Accordingly, different approaches for apple by-product valorisation have been developed in recent years (Table 3).

Chandrasekar and collaborators [100] proved that microwave-assisted extraction, with acetone and ethanol as a solvent system, was an effective technique for recovering antioxidant compounds from apple by-product. More specifically, it was possible to identify phloridzin, caffeic acid, and quercetin for all extracts. Even though this methodology was more efficient as compared with conventional methods, a deeper study was required to determine the feasibility of the process. Bai and collaborators [101] reported a simple and efficient microwave-assisted extraction of polyphenol compounds from apple by-product. Results demonstrated a high extraction yield of 62.68 ± 0.35 mg of gallic acid equivalents per 100 g of dry apple pomace. Furthermore, Dranca, Vargas, and Oraian [102] applied six different non-conventional methodologies for pectin extraction from apple pomace and concluded that the lowest pectin recovery was obtained for the enzymatic Celluclast^®^ 1.5 L extraction, whereas microwave extraction led to the highest extraction yield. Zhang, Poojary, Choudhary, Rai, and Tiwari [103] evaluated microwave extraction for the recovery of phenolic compounds from apple pomace and concluded that the particle size, microwave power level, and irradiation time had a significant influence on phenolics extraction. The higher the microwave power level and the lower the particle size, the greater phenolic compounds were recovered. 

Ultrasound-assisted extraction has proved to increase more than 30% of the total phenolic content in apple pomace, as compared to conventional maceration extraction. Furthermore, in the produced extracts, rich in antioxidants, the main polyphenols were not degraded under the applied conditions [104]. In addition, Egüés and collaborators [105] reported the optimal studied conditions for ultrasound extraction of antioxidant compounds. Thus, conditions were set at 20 min, 90 °C, and 50% ultrasound amplitude. Pollini and collaborators [106] compared different non-conventional techniques, namely ultrasound-assisted extraction, ultra-turrax extraction, accelerated solvent extraction, and pulsed electric field, for the extraction of phenolic compounds. The authors concluded that the highest value of total phenolic content was obtained when ultrasound-assisted extraction was performed. Ultrasound has been utilised also for pectin extraction from apple pomace. Calvete-Torre et al. [107] determined that ultrasound-assisted extraction did not improve the pectin yield, as compared to conventional acid treatment, but the use of ultrasound bath and probe modes allowed the extraction of pectins with modified structural features that could contribute to the broadening food applications of pectin. Zhang and collaborators [103], after studying the ultrasound-assisted and not assisted extraction with pectinase on apple pomace, concluded that ultrasound-assisted enzymatic extraction resulted in the highest recovery of phenolics. The recovery increased with increasing enzyme and decreasing extraction time. Furthermore, a higher enzyme dosage combined with higher sonication levels increased the recovery as well.

Enzymatic-assisted extraction performed with endo-xylanase and endo-cellulase was carried out for the recovery of pectin from apple pomace. Results exhibited an almost complete extraction of pectin and polygalacturonic acid [108]. Furthermore, Gullón and collaborators [109] demonstrated the high susceptibility of apple pomace to enzymatic hydrolysis (cellulase and cellobiase). These authors reported a conversion of about 80% of the total glucan into glucose after 15 h. In addition, apple pomace is a well substrate for fermentation due to its composition properties. Therefore, this biotransformation is a potential way to improve the value of the aforementioned discard [84]. Rodríguez Madrera and collaborators [81] fermented the apple pomace with three yeast strains, namely *S. cerevisiae*, *S. bayanus*, and *H*. *uvarum*. This process enabled a significant increase in protein (23–49%), dietary fibre (30–41%), and fat (17–39%), as well as enhancing the phenolic content and fatty acids proportion. Furthermore, Rodriguez-Madrera et al. [110] increased the nutritional and functional properties of apple by-product through the fermentation of the latter with *S. cerevisiae*. Particularly, the contents of protein (36%) and insoluble dietary fibre (23%) were enhanced. 

Supercritical fluid extraction was explored by Ferrentino et al. [111] to recover antioxidants from apple by-product. Conditions were settled at 45 and 55 °C, whereas pressure was established at 20 and 30 MPa. The extraction was carried out in the absence and presence of ethanol (5%) as a co-solvent. Results demonstrated that the extracts obtained at 30 MPa pressure and 45 °C, with ethanol as a co-solvent, led to a higher antioxidant activity as compared to conventional methods. Woźniak and collaborators [112] proposed SFE as a method for the selective extraction of hydrophobic triterpenic acids and phytosterols. Results demonstrated that 80 °C and 30 MPa of pressure were the optimal conditions. The authors expressed the prospect of this methodology’s industrial scale for the extraction of high-value compounds from apple by-product. SFE employing CO_2_ as a solvent was explored by De la Peña-Armada and collaborators [35] to recover bioactive lipophilic compounds from apple by-product. Besides, there was no co-solvent used to promote a cleaner and safer extraction. The supercritical CO_2_ (scCO_2_) extracts exhibited a desirable antioxidant activity, mainly attributed to fatty acid and triterpenes composition. It was possible to identify oleanolic acid, betulinic acid, ursolic acid, uvaol, erythrodiol, and lupeol, as well as essential fatty acids, namely, palmitic acid, stearic acid, oleic acid, linoleic acid, linolenic acid, and arachidic acid. These valuable components encountered in apple by-product made it a potential antioxidant ingredient. Furthermore, the scCO_2_ extracts were demonstrated to protect human keratinocytes cells by inhibiting the formation of reactive oxygen species (ROS), as well as to enhance mitochondrial activity, and not be toxic. Therefore, the authors concluded that apple by-product supercritical fluid extracts could be of relevance for cosmeceutical formulations. Ferrentino and collaborators [113] investigated the application of SFE to obtain oil from apple seeds and concluded that the recovered oil had high bioactivity, was free from antinutritional compounds, and was suitable for food application. Montañes and collaborators [42] also studied oil extraction from apple seeds and demonstrated that a high-value oil from apple seeds was obtained using ultra-high pressure CO_2_ extraction even though no phenolic compounds were significantly solubilised under any of the extractions.

**Figure 5:**
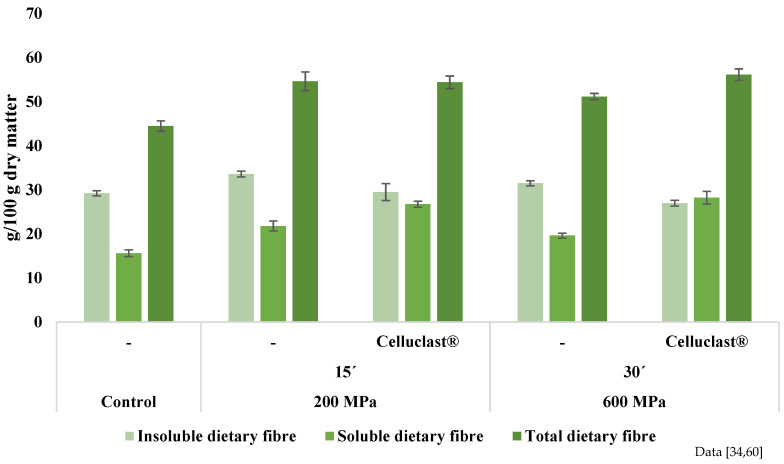
Determination of dietary fibre in apple by-product under high hydrostatic pressure at 200 MPa for 15 min and 600 MPa for 30 min, assisted and not assisted by Celluclast^®^ (92 EGU) enzyme. Data are expressed as g/100 g dry matter.

**Figure 6:**
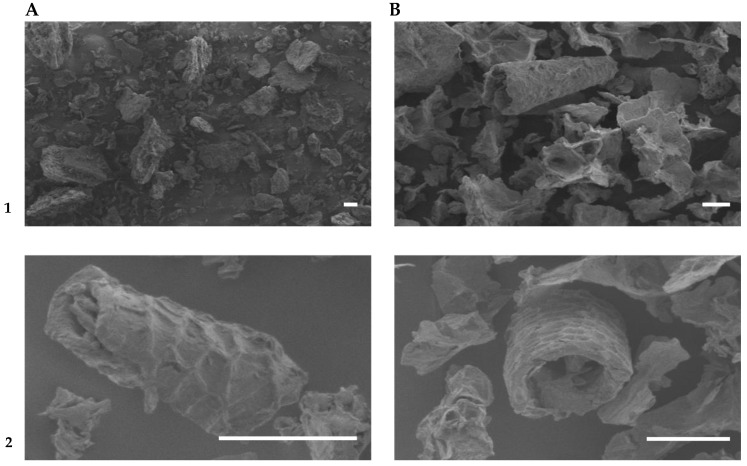
SEM micrographs of samples of apple by-product: control (**A**), HHP-treated at 200 MPa (**B**) (own photos). The white scale bar on micrographs corresponds to 100 μm. Magnification (referred to the 12.67 cm × 8.82 cm raw picture size) was: (a) × 50_(1)_, × 500 _(2),_ (b) × 100_(1)_, × 300_(2)_.

HHP has been demonstrated as an appealing option for apple by-product valorisation. De la Peña-Armada, Mateos-Aparicio, and Villanueva-Suarez [60] assessed the combination of varying time and high pressure for apple by-product processing. The analyses resulted in an increase in the total and soluble dietary fibre fraction (Figure 5), enhancing the solubility of the pectin component for all the tested samples. This effect is related to HHP technology, which can lead to cell wall disruption. As observed by scanning electron microscopy (Figure 6), HHP-treated samples exhibited a less compact and porous microstructure and cavity formation, enabling an increase in cell wall accessibility [34]. Moreover, techno-functional properties were improved, namely, the swelling capacity, oil holding capacity, emulsifying activity, and emulsion stability. Additionally, the high-pressure treatment preserved the sample’s brightness. Hence, HHP seemed to be an interesting green technology for making the apple by-product a more suitable ingredient to be used by the industry in functional food. In addition, De la Peña-Armada and collaborators developed novel procedures for apple by-product valorisation consisting of HHP aided by commercial food-grade enzymes [114]. HHP treatment aided by Celluclast^®^ proved to hydrolyse the insoluble dietary fibre of apple by-product. An increase in the water-soluble polysaccharides (1.8-fold) and oligosaccharides (3.8-fold) was observed, whereas a considerable decrease in the time required to achieve the same results as in absence of Celluclast^®^ enzyme was noted. The study concluded that a potential prebiotic product could be obtained at the lowest pressure tested in a short period of time (200 MPa for 15 min), and thus a promising approach for the valorisation of an insoluble dietary fibre-rich plant by-product was proved. 

## 6. Conclusions

The apple is one of the major fruit crops worldwide. The modern industrial production of apple-based beverages generates hundreds of tonnes of apple by-product yearly, which are commonly discarded as waste. Nonetheless, this derived product is considered a natural source of bioactive compounds with great potential beneficial effects for human health. The application of green technologies on apple by-product provides a novel approach for apple by-product valorisation in order to preserve and promote its functionality and nutritional value. Different techniques are presented to compare conditions and determine the main target bioactive compound achievement through the exposed methodologies. After the application of HHP, or assisted extraction with enzymes, supercritical fluids, microwaves, or ultrasounds, it seems possible to transform this perishable material into an interesting source of health-promoting compounds, such as dietary fibre, oligosaccharides, and antioxidants. Thus, the apple, which has played an important role throughout human history, may be fully exploited and extended to the currently underestimated apple by-product, which may become a newly added value product. 

## Figures and Tables

**Table 1:** Proximate composition of apple pomace in dry matter (%).

TDF	SDF	IDF	Non-Fibrous Carbohydrates	Protein	Lipids	Ashes	Reference
55.48 ± 0.7	11.06 ± 0.1	43.58 ± 0.6	28.48 ± 1.1	6.25 ± 0.1	6.58 ± 0.1	1.56 ± 0.3	[45]
-	-	-	-	3.8 ± 0.13	1.2 ± 0.10	1.5 ± 0.02	[46]
-	20.63 ± 2.00	62.08 ± 0.77	-	4.36 ± 0.24	7.36 ± 0.74	-	[47]
64.84 ± 1.78	20.27 ± 0.09	44.57 ± 0.24	-	3.57 ± 0.08	-	4.29 ± 0.06	[48]
53.1 ± 0.7	6.1 ± 0.2	47.0 ± 0.8	-	0.5 ± 0.0	-	1.8 ± 0.0	[49]
76.84 ± 1.24	18.97 ± 0.92	57.87 ± 0.33	6.72 ± 1.54	6.98 ± 0.32	8.19 ± 0.05	1.26 ± 0.12	[50]
60.48	-	-	-	2.28	3.84	6.67	[51]
8.86 ± 0.50			74.44 ± 0.15	3.42 ± 0.94	0.26 ± 0.13	1.68 ± 0.74	[52]
51.10 ± 1.86	14.60 ± 0.14	36.50 ± 1.14	-	2.06 ± 0.05	2.70 ± 0.10	0.50 ± 0.05	[53]
78.2 ± 0.60	14.33 ± 0.61	63.9 ± 0.16	13.3	3.12 ± 0.07	1.57 ± 0.08	1.88 ± 0.11	[54]

TDF: total dietary fibre; SDF: soluble dietary fibre; IDF: insoluble dietary fibre.

**Table 2:** Revision of the studies of the effects of the apple by-product on health.

Study Characteristics	Results	References
Female Sprague–Dawley rats randomly assigned: standard purified rodent diet or AIN-93G, AIN-93G with freeze-dried apple pomace, or Western diet, or Western diet with freeze-dried apple pomace.	Improved liver and adipose inflammatory and antioxidant status: Rats consuming Western/AP downregulated hepatic and adipose proinflammatory cytokine gene expression and improved antioxidant status compared to rats consuming a Western diet.	[55]
In vitro digested apple pomace and pectin fractions derived from three apple varieties were subjected to faecal batch fermentation by using samples from healthy donors and from patients of Crohn’s disease.	Prebiotic potential: Growth of Akkermansia, Lachnospiraceae UCG-010, Prevotella, Sucinivibrio, and Turicibacter on samples from healthy donors, whereas Blautia, Lachnospiraceae CAG-56, Dialister, Eubacterium eligens, and Intestinimonas were stimulated in fermentations from inflammatory bowel disease patients.	[56]
Male C57BL/6J mice were exposed to a high-fat and sucrose diet without and with the addition of 10 mg apple pomace flower per day whereas the control groups were fed with standard pellet rodent diet without.	Antioxidant, antidiabetic, and antiobesity effects: Long-term supplementation with apple pomace flower was shown to decrease glycemia, significantly improve glucose tolerance, and decrease body weight gain in mice exposed to a high-fat and sucrose diet.	[57]
Wistar Hannover rats were fed with high-fat diets for 5 weeks and randomised in two groups (control and supplemented with apple by-product). Diets were prepared from a commercial diet AIN-210 enriched with fat.	Prebiotic and hypolipidemic effect: Butyrate was increased 3-fold in this in vivo assay indicating that butyrate bacteria producers can use apple by-product. Apple by-product enriched diet increased HDL and diminished trygliceridemia and hepatic total lipids.	[45]
Male F344 rats were fed a control feed or the same feed with 2.1%, or 6.5% dry apple pomace, with or without seeds for 4 weeks.	Hypocholesterolemic effect and improved gut health: Pomace feeding decreased total-, LDL- and IDL-cholesterol concentrations, increased production of SCFA and increased excretion of total- and primary bile acids. No hepatotoxic or other effects were derived from apple seeds.	[58]
Male Sprague–Dawley rats were assigned to four groups: the normal diet group, the high-fat diet group, and high-fat diet group containing either apple pomace or apple juice concentrate.	Loss of body weight and fat and improved lipid profiles: Body weight gain, white adipose tissue, weight, serum total cholesterol, LDL cholesterol, and triglyceride concentrations, epididymal adipocyte size, and lesion scores were significantly lower and serum HDL cholesterol concentration and brown adipose tissue weights were significantly higher in the apple pomace and apple juice concentrate groups compared with the high-fat diet group.	[59]

**Table 3:** Summary of the sustainable treatments applied to apple by-product.

Extraction Methodology	Application	Conditions Tested	References
Microwave	Antioxidant compound recovery	Solvent type: 70% acetone and 60% ethanol Microwave power: 100–900 W Solvent volume to sample ratio: 4–12 mL/g dry pomace. Extraction time: 30–180 s	[100]
	Polyphenol extraction	Microwave power: 500–700 W Extraction time: 40–60 s Ethanol concentration: 50–70% Ratio of solvent to raw material: 10:1–30:1 mL/g	[101]
	Pectin extraction	Microwave power: 560 W Extraction time: 120 s	[102]
	Phenolic compound extraction	Microwave power: 400, 600, 1000 W Extraction time: 60, 90 s	[103]
Ultrasound	Polyphenol extraction	UI: 0.431–0.764 W/cm^2^ Temperature: 16–40 °C Sonication time: 5–55 min	[104]
	Extraction of antioxidant compounds	Amplitude: 50–70% Temperature: 40–90 °C Sonication time: 5–20 min	[105]
	Phenolic compounds extraction	EtOH:H_2_O ratios: 50:50, 70:30, 30:70, *v*/*v* Liquid/solid ratio: 1:10 g/mL Time: 60 min Temperature: 60 °C	[106]
	Pectin extraction	Mode: bath, probe Time: 30, 60 min Frequency ultrasonic bath: 45 kHz Temperature: 60 °C Amplitude probe mode: 30%, 50%	[107]
	Phenolic compound extraction	Time: 2, 5, 10, 20, 30 min Temperature: 25 °C Sonication powers: 7.8, 49.5 W	[103]
	Phenolic compound extraction	Time: 2, 5, 10, 20, 30 min Temperature: 25 °C Sonication powers: 7.8, 49.5 W Enzyme: pectinase	[103]
Enzymatic	Pectin recovery	Endo-xylanase and endo-cellulase Extraction time: 10 h Temperature: 40 °C pH 5.0	[108]
	Raw material	Enzyme concentrates: Celluclast 1.5 L and b-glucosidase Temperature: 48.5 °C Orbital agitation: 150 rpm pH: 4.85	[109]
	Nutritional composition and phenolic compounds	Three yeast strains: *S. cerevisiae*; *S. bayanus,* and *H. uvarum*. Time: 7 days Temperature: 25 °C	[81]
	Nutritional and functional properties	Yeast strain: *S. cerevisiae* Time: 4.9 days Temperature: 29.5 °C	[110]
Supercritical CO_2_	Extraction of antioxidants	Pressure: 20–30 bar Temperature: 45–55 °C Co-solvent: ethanol (5%)	[111]
	Extraction of Triterpenic acids and phytosterols	Pressure: 20–30 bar Temperature: 60–80 °C Flow rate: 4.17–12,5 *10^−4^ L s^−1^	[112]
	Recover bioactive lipophilic compounds	Pressure: 300–550 bar Temperature: 37–55 °C Flow rate: 10 g/min	[35]
	Oil extraction	Pressure: 10–30 bar Temperature: 40–60 °C Flow rate: 1–8 L/h	[113]
	Oil extraction	Pressure: 300–1300 bar Temperature: 316–336 K Flow rate: 6–10 mL/min	[42]
High hydrostatic pressure	Pectin recovery	Pressure: 200–600 MPa Temperature: 50 °C Time: 15–30 min	[60]
	Pectin and oligosaccharides recovery	Pressure: 200–600 MPa Enzyme: 92 EGU Celluclast^®^ Temperature: 50 °C Time: 15–30 min	[34]

s: second; min: minutes; h: hour; W: watt; W/cm^2^: watt per square centimetre; rpm: revolution per minute; MPa: megapascal; EGU: endo-glucanase units.

## Data Availability

Not applicable.
